# (2*E*)-1-(5-Bromothiophen-2-yl)-3-(4-chloro­phen­yl)prop-2-en-1-one

**DOI:** 10.1107/S1600536813012063

**Published:** 2013-05-11

**Authors:** H. D. Kavitha, K. R. Roopashree, Suresh B. Vepuri, H. C. Devarajegowda, Venkatesh B. Devaru

**Affiliations:** a Department of Physics, Govt. Science College, Hassan 573 201, Karnataka, India; b Department of Physics, Yuvaraja’s College (Constituent College), University of Mysore, Mysore 570005, Karnataka, India; c Institute of Pharmacy, GITAM University, Visakhapatnam-45, Andhrapradesh, India; d Department of Physics, P. G. Department of Physics, LVD College, Raichur 584103, Karnataka, India

## Abstract

In the title compound, C_13_H_8_BrClOS, the thio­phene and phenyl rings are inclined by 40.69 (11)° to each other. The crystal structure is characterized by C—H⋯π inter­actions, which link the mol­ecules into broad layers parallel to (100). Short Br⋯Cl contacts [3.698 (1) Å] link these layers along [100].

## Related literature

For general background to chalcones, see: Chun *et al.* (2001 ▶); Horng *et al.* (2003 ▶); Lopez *et al.* (2001 ▶); Mei *et al.* (2003 ▶). For related structures, see: Vepuri *et al.* (2012 ▶); Li & Su (1993 ▶).
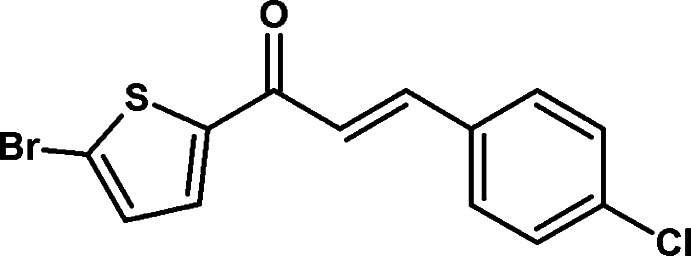


## Experimental

### 

#### Crystal data

C_13_H_8_BrClOS
*M* *_r_* = 327.61
Monoclinic, 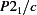

*a* = 15.235 (3) Å
*b* = 13.959 (3) Å
*c* = 5.9153 (11) Å
β = 93.259 (3)°
*V* = 1255.9 (4) Å^3^
*Z* = 4
Mo *K*α radiation
μ = 3.63 mm^−1^
*T* = 293 K
0.24 × 0.20 × 0.12 mm

#### Data collection

Bruker SMART CCD area-detector diffractometer
Absorption correction: multi-scan (*SADABS*; Sheldrick, 2007 ▶) *T*_min_ = 0.770, *T*_max_ = 1.000
14247 measured reflections
3032 independent reflections
2204 reflections with *I* > 2σ(*I*)
*R*_int_ = 0.023

#### Refinement

*R*[*F* ^2^ > 2σ(*F* ^2^)] = 0.030
*wR*(*F* ^2^) = 0.081
*S* = 1.05
3032 reflections
154 parameters
H-atom parameters constrained
Δρ_max_ = 0.40 e Å^−3^
Δρ_min_ = −0.29 e Å^−3^

### 

Data collection: *SMART* (Bruker, 2001 ▶); cell refinement: *SAINT* (Bruker, 2001 ▶); data reduction: *SAINT*; program(s) used to solve structure: *SHELXS97* (Sheldrick, 2008 ▶); program(s) used to refine structure: *SHELXL97* (Sheldrick, 2008 ▶); molecular graphics: *ORTEP-3 for Windows* (Farrugia, 2012 ▶); software used to prepare material for publication: *SHELXL97*.

## Supplementary Material

Crystal structure: contains datablock(s) I, global. DOI: 10.1107/S1600536813012063/bg2505sup1.cif

Structure factors: contains datablock(s) I. DOI: 10.1107/S1600536813012063/bg2505Isup2.hkl

Supplementary material file. DOI: 10.1107/S1600536813012063/bg2505Isup3.cml

Additional supplementary materials:  crystallographic information; 3D view; checkCIF report

## Figures and Tables

**Table 1:** Hydrogen-bond geometry (Å, °) *Cg*2 is the centroid of the C5–C10 ring.

*D*—H⋯*A*	*D*—H	H⋯*A*	*D*⋯*A*	*D*—H⋯*A*
C10—H10⋯*Cg*2^i^	0.93	2.87	3.557 (3)	132
C16—H16⋯*Cg*2^ii^	0.93	2.96	3.480 (3)	117

Symmetry codes: (i) 
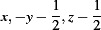
; (ii) 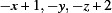.

**Fig. 1.**
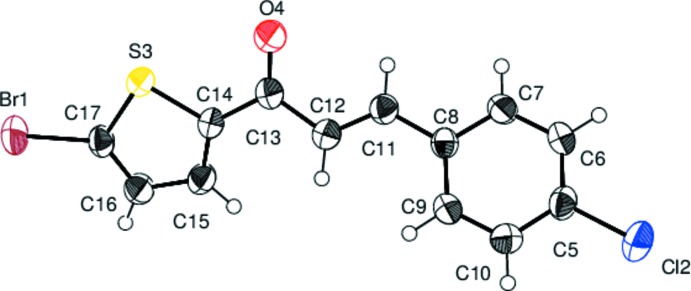
The molecular structure of the title compound. Displacement ellipsoids are drawn at the 50% probability level.

**Fig. 2.**
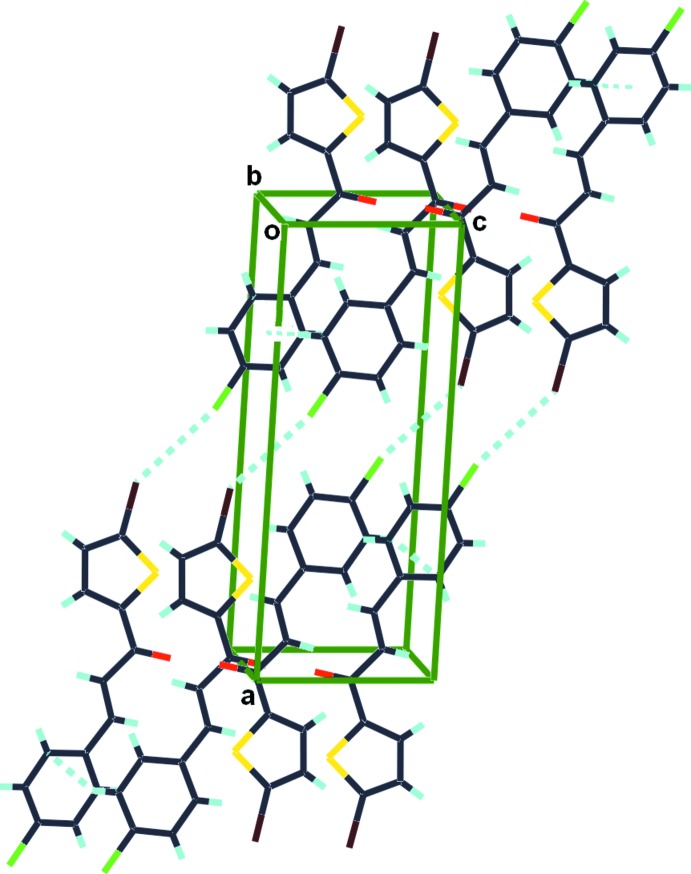
Crystal packing of the title compound. Dashed lines represent C-H···π and Br···Cl bonds.

## supplementary crystallographic information

### Comment

Chalcones are alpha beta unsaturated ketones, widely distributed in nature and are extensively studied for their biological activity (Chun *et al.*, 2001; Horng *et al.*, 2003; Lopez *et al.*, 2001; Mei *et al.*, 2003). We report here the crystal structure of a bromo derivative of hetero aryl chalcone which has shown aldose reductase inhibition in the virtual screening study conducted by us.

The titlecompound (2E)-1-(5-bromo-2-thienyl)-3-(4- chlorophenyl)prop-2-en-1-one, C_13_H_8_Br Cl O S, presents a five-membered thiophene ring (S3\C14\···C17) and a phenyl ring (C5\C6\···C10) at 40.69 (11)° to each other (Fig 1). All intermolecular bond lengths and angles are within normal ranges (Vepuri *et al.*, 2012; Li & Su, 1993). The crystal structure is characterized by C—H···π interactions (C10—H10···Cg2; C16—H16···Cg2, Cg2 = C5->C10) (Table 1) which link molecules into broad 2D structures parallel to (100). There are in addition short intermolecular Br1···Cl2 contacts of 3.698 (1) Å, which link these structures along [100]. (Fig 2)

### Experimental

A mixture of 2-acetyl-5-BromoThiophene (0.01 mole) and 4-chlorobenzaldehyde (0.01 mole) were stirred in ethanol (30 ml) and then an aqueous solution of potassium hydroxide (40%,15 ml)was added to it. The mixture was kept over night at room temperature and then it was poured into crushed ice and acidified with dilute hydrochloric acid. The precipiteted chalcone was filtered and crystallized from ethanol.

### Refinement

All H atoms were positioned geometrically, with C—H = 0.93 Å for aromatic H and refined using a riding model with *U*_iso_(H) = 1.2*U*_eq_(C) for aromatic H.

### Crystal data

**Table:**

C_13_H_8_BrClOS	*F*(000) = 648
*M**_r_* = 327.61	*D*_x_ = 1.733 Mg m^−^^3^
Monoclinic, *P*2_1_/*c*	Melting point: 399 K
Hall symbol: -P 2ybc	Mo *K*α radiation, λ = 0.71073 Å
*a* = 15.235 (3) Å	Cell parameters from 2202 reflections
*b* = 13.959 (3) Å	θ = 2.0–25.0°
*c* = 5.9153 (11) Å	µ = 3.63 mm^−^^1^
β = 93.259 (3)°	*T* = 293 K
*V* = 1255.9 (4) Å^3^	Plate, colourless
*Z* = 4	0.24 × 0.20 × 0.12 mm

### Data collection

**Table:**

Bruker SMART CCD area-detector diffractometer	3032 independent reflections
Radiation source: fine-focus sealed tube	2204 reflections with *I* > 2σ(*I*)
Graphite monochromator	*R*_int_ = 0.023
ω and φ scans	θ_max_ = 25.0°, θ_min_ = 2.0°
Absorption correction: multi-scan (*SADABS*; Sheldrick, 2007)	*h* = −18→18
*T*_min_ = 0.770, *T*_max_ = 1.000	*k* = 0→16
14247 measured reflections	*l* = 0→7

### Refinement

**Table:**

Refinement on *F*^2^	Primary atom site location: structure-invariant direct methods
Least-squares matrix: full	Secondary atom site location: difference Fourier map
*R*[*F*^2^ > 2σ(*F*^2^)] = 0.030	Hydrogen site location: inferred from neighbouring sites
*wR*(*F*^2^) = 0.081	H-atom parameters constrained
*S* = 1.05	*w* = 1/[σ^2^(*F*_o_^2^) + (0.0473*P*)^2^ + 0.2986*P*] where *P* = (*F*_o_^2^ + 2*F*_c_^2^)/3
2202 reflections	(Δ/σ)_max_ = 0.001
154 parameters	Δρ_max_ = 0.40 e Å^−^^3^
0 restraints	Δρ_min_ = −0.29 e Å^−^^3^

### Special details

**Table:**

Geometry. All s.u.'s (except the s.u. in the dihedral angle between two l.s. planes) are estimated using the full covariance matrix. The cell s.u.'s are taken into account individually in the estimation of s.u.'s in distances, angles and torsion angles; correlations between s.u.'s in cell parameters are only used when they are defined by crystal symmetry. An approximate (isotropic) treatment of cell s.u.'s is used for estimating s.u.'s involving l.s. planes.
Refinement. Refinement of *F*^2^ against ALL reflections. The weighted *R*-factor *wR* and goodness of fit *S* are based on *F*^2^, conventional *R*-factors *R* are based on *F*, with *F* set to zero for negative *F*^2^. The threshold expression of *F*^2^ > 2σ(*F*^2^) is used only for calculating *R*-factors(gt) *etc*. and is not relevant to the choice of reflections for refinement. *R*-factors based on *F*^2^ are statistically about twice as large as those based on *F*, and *R*- factors based on ALL data will be even larger.

### Fractional atomic coordinates and isotropic or equivalent isotropic displacement parameters (Å^2^)

**Table:**

	*x*	*y*	*z*	*U*_iso_*/*U*_eq_	
Br1	0.876810 (18)	0.34860 (2)	0.10591 (6)	0.06662 (15)	
Cl2	0.03637 (5)	0.36156 (7)	0.68667 (14)	0.0719 (3)	
S3	0.67904 (4)	0.34879 (5)	−0.04338 (10)	0.04481 (18)	
O4	0.48985 (13)	0.37196 (16)	−0.1517 (3)	0.0642 (6)	
C5	0.13007 (16)	0.37433 (18)	0.5358 (4)	0.0436 (6)	
C6	0.12401 (15)	0.41523 (19)	0.3238 (4)	0.0466 (6)	
H6	0.0705	0.4381	0.2626	0.056*	
C7	0.19854 (15)	0.42171 (17)	0.2044 (4)	0.0421 (6)	
H7	0.1943	0.4480	0.0598	0.051*	
C8	0.28016 (15)	0.39015 (16)	0.2928 (4)	0.0369 (5)	
C9	0.28356 (16)	0.35027 (16)	0.5098 (4)	0.0407 (6)	
H9	0.3372	0.3292	0.5743	0.049*	
C10	0.20961 (17)	0.34157 (16)	0.6295 (4)	0.0431 (6)	
H10	0.2129	0.3138	0.7727	0.052*	
C11	0.35582 (15)	0.39464 (17)	0.1545 (4)	0.0399 (5)	
H11	0.3445	0.4112	0.0034	0.048*	
C12	0.43893 (16)	0.37794 (19)	0.2184 (4)	0.0450 (6)	
H12	0.4544	0.3669	0.3706	0.054*	
C13	0.50768 (17)	0.37658 (18)	0.0522 (4)	0.0431 (6)	
C14	0.59921 (15)	0.37793 (16)	0.1398 (4)	0.0371 (5)	
C15	0.63572 (16)	0.40178 (19)	0.3477 (4)	0.0454 (6)	
H15	0.6027	0.4194	0.4684	0.055*	
C16	0.72770 (16)	0.39728 (19)	0.3626 (4)	0.0464 (6)	
H16	0.7625	0.4121	0.4922	0.056*	
C17	0.75950 (15)	0.36879 (17)	0.1654 (4)	0.0410 (6)	

### Atomic displacement parameters (Å^2^)

**Table:**

	*U*^11^	*U*^22^	*U*^33^	*U*^12^	*U*^13^	*U*^23^
Br1	0.03415 (18)	0.0911 (3)	0.0755 (2)	0.00978 (13)	0.01087 (14)	0.00945 (16)
Cl2	0.0432 (4)	0.1058 (7)	0.0687 (5)	−0.0075 (4)	0.0196 (3)	0.0045 (4)
S3	0.0374 (4)	0.0601 (4)	0.0373 (3)	0.0041 (3)	0.0062 (3)	−0.0034 (3)
O4	0.0443 (11)	0.1042 (17)	0.0438 (11)	0.0027 (10)	−0.0004 (8)	−0.0067 (10)
C5	0.0348 (13)	0.0501 (14)	0.0466 (14)	−0.0074 (11)	0.0069 (11)	−0.0050 (12)
C6	0.0318 (13)	0.0592 (17)	0.0483 (15)	0.0033 (11)	−0.0024 (10)	0.0017 (12)
C7	0.0377 (13)	0.0474 (14)	0.0407 (13)	0.0003 (11)	−0.0025 (10)	0.0034 (11)
C8	0.0344 (12)	0.0350 (12)	0.0411 (13)	−0.0037 (10)	0.0003 (10)	−0.0021 (10)
C9	0.0351 (13)	0.0433 (14)	0.0431 (14)	0.0022 (10)	−0.0029 (11)	−0.0003 (10)
C10	0.0452 (14)	0.0432 (14)	0.0408 (14)	−0.0018 (11)	0.0013 (11)	0.0006 (10)
C11	0.0367 (13)	0.0429 (13)	0.0400 (13)	−0.0018 (10)	0.0020 (10)	−0.0024 (10)
C12	0.0369 (14)	0.0580 (15)	0.0402 (13)	0.0007 (11)	0.0039 (11)	0.0015 (11)
C13	0.0367 (13)	0.0484 (14)	0.0441 (15)	0.0010 (11)	0.0017 (11)	0.0003 (11)
C14	0.0320 (12)	0.0393 (13)	0.0406 (13)	0.0014 (10)	0.0077 (10)	−0.0008 (10)
C15	0.0424 (14)	0.0525 (16)	0.0420 (14)	0.0038 (11)	0.0077 (11)	−0.0078 (11)
C16	0.0407 (14)	0.0555 (16)	0.0430 (14)	−0.0036 (12)	0.0004 (11)	−0.0066 (12)
C17	0.0300 (12)	0.0426 (13)	0.0507 (14)	−0.0018 (10)	0.0042 (10)	0.0034 (11)

### Geometric parameters (Å, º)

**Table:**

Br1—C17	1.863 (2)	C9—C10	1.370 (3)
Cl2—C5	1.735 (3)	C9—H9	0.9300
S3—C17	1.713 (3)	C10—H10	0.9300
S3—C14	1.723 (2)	C11—C12	1.321 (3)
O4—C13	1.223 (3)	C11—H11	0.9300
C5—C6	1.376 (4)	C12—C13	1.477 (3)
C5—C10	1.381 (4)	C12—H12	0.9300
C6—C7	1.374 (3)	C13—C14	1.460 (3)
C6—H6	0.9300	C14—C15	1.362 (3)
C7—C8	1.393 (3)	C15—C16	1.400 (3)
C7—H7	0.9300	C15—H15	0.9300
C8—C9	1.397 (3)	C16—C17	1.348 (3)
C8—C11	1.452 (3)	C16—H16	0.9300
			
C17—S3—C14	90.57 (12)	C12—C11—H11	116.2
C6—C5—C10	121.0 (2)	C8—C11—H11	116.2
C6—C5—Cl2	119.8 (2)	C11—C12—C13	121.1 (2)
C10—C5—Cl2	119.2 (2)	C11—C12—H12	119.5
C7—C6—C5	118.8 (2)	C13—C12—H12	119.5
C7—C6—H6	120.6	O4—C13—C14	120.3 (2)
C5—C6—H6	120.6	O4—C13—C12	122.1 (2)
C6—C7—C8	122.2 (2)	C14—C13—C12	117.6 (2)
C6—C7—H7	118.9	C15—C14—C13	131.0 (2)
C8—C7—H7	118.9	C15—C14—S3	111.04 (18)
C7—C8—C9	117.2 (2)	C13—C14—S3	117.92 (18)
C7—C8—C11	119.7 (2)	C14—C15—C16	113.7 (2)
C9—C8—C11	123.1 (2)	C14—C15—H15	123.2
C10—C9—C8	121.4 (2)	C16—C15—H15	123.2
C10—C9—H9	119.3	C17—C16—C15	111.5 (2)
C8—C9—H9	119.3	C17—C16—H16	124.3
C9—C10—C5	119.4 (2)	C15—C16—H16	124.3
C9—C10—H10	120.3	C16—C17—S3	113.23 (19)
C5—C10—H10	120.3	C16—C17—Br1	127.2 (2)
C12—C11—C8	127.6 (2)	S3—C17—Br1	119.61 (14)
			
C10—C5—C6—C7	1.0 (4)	C11—C12—C13—C14	167.3 (2)
Cl2—C5—C6—C7	−177.9 (2)	O4—C13—C14—C15	164.8 (3)
C5—C6—C7—C8	−1.4 (4)	C12—C13—C14—C15	−17.0 (4)
C6—C7—C8—C9	0.6 (4)	O4—C13—C14—S3	−12.8 (3)
C6—C7—C8—C11	177.1 (2)	C12—C13—C14—S3	165.31 (18)
C7—C8—C9—C10	0.7 (3)	C17—S3—C14—C15	0.3 (2)
C11—C8—C9—C10	−175.7 (2)	C17—S3—C14—C13	178.47 (19)
C8—C9—C10—C5	−1.1 (4)	C13—C14—C15—C16	−177.6 (2)
C6—C5—C10—C9	0.2 (4)	S3—C14—C15—C16	0.2 (3)
Cl2—C5—C10—C9	179.09 (18)	C14—C15—C16—C17	−0.8 (3)
C7—C8—C11—C12	171.9 (3)	C15—C16—C17—S3	1.0 (3)
C9—C8—C11—C12	−11.8 (4)	C15—C16—C17—Br1	−177.94 (19)
C8—C11—C12—C13	174.2 (2)	C14—S3—C17—C16	−0.8 (2)
C11—C12—C13—O4	−14.5 (4)	C14—S3—C17—Br1	178.26 (15)

### Hydrogen-bond geometry (Å, º)

Cg2 is the centroid of the C5–C10 ring.

**Table:**

*D*—H···*A*	*D*—H	H···*A*	*D*···*A*	*D*—H···*A*
C10—H10···*Cg*2^i^	0.93	2.87	3.557 (3)	132
C16—H16···*Cg*2^ii^	0.93	2.96	3.480 (3)	117

Symmetry codes: (i) *x*, −*y*−1/2, *z*−1/2; (ii) −*x*+1, −*y*, −*z*+2.
